# Intraoperative radiotherapy after neurosurgical resection of brain metastases as institutional standard treatment– update of the oncological outcome form a single center cohort after 117 procedures

**DOI:** 10.1007/s11060-024-04691-6

**Published:** 2024-07-04

**Authors:** Klaus-Henning Kahl, Philipp E. Krauss, Maria Neu, Christoph J. Maurer, Sabine Schill-Reiner, Zoha Roushan, Eva Laukmanis, Christian Dobner, Tilman Janzen, Nikolaos Balagiannis, Björn Sommer, Georg Stüben, Ehab Shiban

**Affiliations:** 1 Department of Radiotherapy and Radio- Oncology, University Medical Center Augsburg, Augsburg, Germany; 2 Department of Neurosurgery, University Medical Center Augsburg, Augsburg, Germany; 3 Department of Diagnostic and Interventional Radiology and Neuroradiology, University Medical Center Augsburg, Augsburg, Germany; 4 Department of Medical Physics and Radiation Protection, University Medical Center Augsburg, Augsburg, Germany

**Keywords:** IORT, Brachytherapy, Cavity RT, Focal radiotherapy, Brain metastasis

## Abstract

**Purpose:**

Stereotactic radiotherapy (SRT) is the predominant method for the irradiation of resection cavities after resection of brain metastases (BM). Intraoperative radiotherapy (IORT) with 50 kV x-rays is an alternative way to irradiate the resection cavity focally. We have already reported the outcome of our first 40 IORT patients treated until 2020. Since then, IORT has become the predominant cavity treatment in our center due to patients´ choice.

**Methods:**

We retrospectively analyzed the outcomes of all patients who underwent resection of BM and IORT between 2013 and August 2023 at Augsburg University Medical Center (UKA).

**Results:**

We identified 105 patients with 117 resected BM treated with 50 kV x-ray IORT. Median diameter of the resected metastases was 3.1 cm (range 1.3 – 7.0 cm). Median applied dose was 20 Gy. All patients received standardized follow-up (FU) including three-monthly MRI of the brain. Mean FU was 14 months, with a median MRI FU for patients alive of nine months. Median overall survival (OS) of all treated patients was 18.2 months (estimated 1-year OS 57.7%). The observed local control (LC) rate of the resection cavity was 90.5% (estimated 1-year LC 84.2%). Distant brain control (DC) was 61.9% (estimated 1-year DC 47.9%). Only 16.2% of all patients needed WBI in the further course of disease. The observed radio necrosis rate was 2.6%.

**Conclusion:**

After 117 procedures IORT still appears to be a safe and appealing way to perform cavity RT after neurosurgical resection of BM with low toxicity and excellent LC.

**Supplementary Information:**

The online version contains supplementary material available at 10.1007/s11060-024-04691-6.

## Introduction

Surgical resection is the preferred treatment of large and symptomatic brain metastases (BM)[1–3]. Due to high local recurrence rates after surgery alone, additional radiotherapy (RT) is necessary to achieve optimal local control (LC) [4]. In this setting whole brain irradiation (WBI) has been replaced by focal RT of the resection cavity because of favorable long term neurotoxicity rates [5, 6]. Stereotactic radiotherapy (SRT) is the predominant method for the irradiation of resection cavities after resection of brain metastases (BM)[7]. Intraoperative radiotherapy (IORT) with 50 kV x-rays is an alternative way to irradiate the resection cavity focally [8, 9]. This method holds the advantage of steeper dose gradients compared to SRT[10, 11]. This leads to a lesser treated volume of brain tissue, which is likely to transform into lower radio necrosis rates with improved LC. From a radio-biological point of view 50 kV x-rays have a higher relative biological efficacy compared to 6MV photons. This means 1 Gy of 50 kV X-rays has the same biological effect as approximately 1.3 Gy of 6MV photons. Furthermore IORT shortens the interval between surgery and radiotherapy to literally zero. This interval is known to be an important factor influencing LC in several settings of adjuvant RT[12]. Finally there are hints that high focal doses of IORT modulate the immunologic response to tumor surgery in a favorable way, enhancing antitumor immune responses[13, 14].

We have already reported the outcome of our first 40 IORT patients treated until 2020[15]. Since then, IORT has become the predominant cavity treatment in our center due to patients´ choice. In the last two and a half years until August 2023 we treated more patients with IORT after BM resection than within the eight years period of our first publication. This rose the question whether the use of IORT after resection of BM in a high-volume routine setting would have an impact on the oncological outcome and toxicity of this procedure.

## Material & methods

We conducted a retrospective analysis of all patients, who were treated with IORT after neurosurgical resection of BM between January 2013 and August 2023 at UKA. The study protocol was approved by the local ethics committee in accordance to the Declaration of Helsinki. For this retrospective observational study, no individual informed consent was necessary according to the ethics committee’s guidelines and regulations. We identified all patients from our oncology information system MOSAIQ (ELEKTA AB, Stockholm) and gained additional information via the hospital information system ORBIS (DEDALUS Healthcare Group AG, Bonn) and the radiology information and picture archiving and communication system Deep Unity (DEDALUS Healthcare Group AG, Bonn). The time point for the last FU included in this analysis was October 18th 2023.

Treatment of all cases followed the recommendations of UKA multidisciplinary tumor board (MTB). With regard to patient selection, a minimal distance of 5 mm between the border of the contrast enhancing lesion in MRI and the optic tract/brainstem was mandatory. Depending on the decision of the neurosurgeon some, but not all patients with centrally located metastases or metastases in the posterior fossa have been excluded. After informed consent of the patient microsurgical BM resection was performed including a frozen section to confirm malignancy of the removed tumor. Hereafter the resection cavity was irradiated with 50 kV x-rays via an INTRABEAM system (ZEISS MEDITEC AG, Oberkochen) equipped with spherical applicators. The device and procedure has already been described before [8, 16]. Spherical applicator sizes of this IORT system range from 15 to 50 mm in diameter in 5 mm increments. The suitable applicator size was chosen by neurosurgeon and radio-oncologist corresponding to the size of the resection cavity, providing direct contact of the cavity walls to the surface of the applicator. Radiation dose was prescribed to the surface of the applicator (tissue depth 0 mm), corresponding to the target volume/ dose concept of postoperative SRT cavity treatment (GTV = CTV = cavity). Due to the dose distribution of the system a 2 mm rim around the cavity received between 63 and 84% of the prescribed dose depending on the size of the applicator. The applied dose is reduced to 38–53% in 5 mm and 18–32% in 10 mm tissue depth. After IORT, the applicator was removed and surgery was completed. After treatment, all patients received standardized FU including 3-monthly MRI of the brain, according to UKA FU policy for SRT. All statistical analyses of this article were performed with EZR (Version 3.4.1 /The R Foundation for statistical computing)[17] using Kaplan–Meier methods and log rank tests.

## Results

**Fig. 1:**
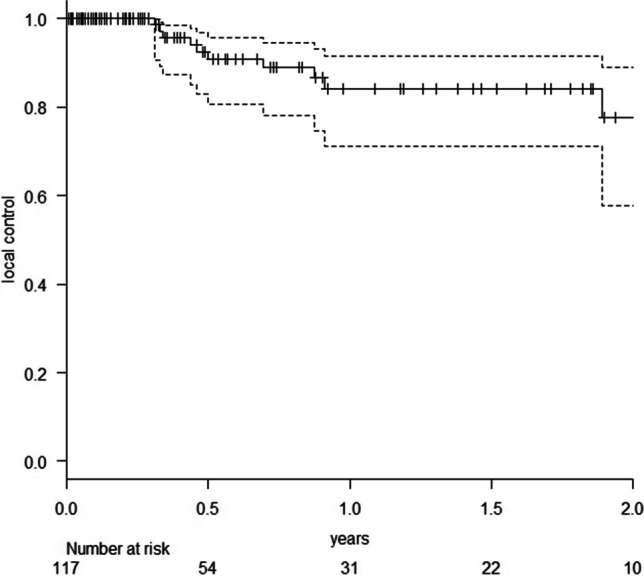
Probability of local control after resection of BM and IORT (the dotted lines represent the 95% confidence intervals)

We identified 105 patients (55 female/ 50 male) with 117 resected BM, who were treated with 50 kV x-ray IORT. For patients characteristics see Table 1. Median age of these patients at time of treatment was 65 years (range 39—88 years). Most patients fitted to recursive partitioning analysis (RPA) [18] class 2 (76 pts. / class 1: 16 pts. / class 3: 13 pts.). Median diameter of the resected metastases was 3.1 cm (range 1.3 – 7.0 cm). Median applied dose was 20 Gy (range 13.4 – 30 Gy). Four patients had a history of previous external beam radiotherapy in the area of resection. Five other patients had received focal RT to BM distant to the resection area before. All other patients were newly diagnosed with BM prior to resection. Median number of BM at treatment time was one (range 1–6). Maximum number of IORT procedures per patient was two. All other non-resected brain lesions were treated with SRT with exception of one patient receiving additional WBI. The predominant histology of the resected metastases was non-small-cell lung cancer (50 metastases), followed by malignant melanoma (16 metastases) and breast carcinoma (13 metastases). Sixty-five of all patients were simultaneously suffering from additional tumor burden in other organs than brain. Mean FU was 14 months (SD: 18 months), with a median MRI FU for patients alive of 9 months (range 0–79 months). At the time of this analysis 52 of these 105 patients had died. Median overall survival of all treated patients was 18.2 months (range 0.5 -79.0 months) with an estimated overall survival at 1 year of 57.7% (95% CI: 46.6–67.4%). The observed LC rate of the resection cavity was 90.5% with estimated LC (Fig. 1) of 84.2% at 1 year (95% CI: 71.3–91.6%). All recurrences despite one were histologically proven (3xNSCLC, breast cancer, malignant melanoma, ovarian cancer, colorectal cancer, esophageal cancer, renal cell cancer and bladder cancer). Observed distant brain control (DC) was 61.9% with estimated DC of 47.9% at 1 year (95% CI: 34.7–60.0%), including six patients (5.7%), who developed leptomeningeal disease (LMD). The estimated LMD rate was 10.4% at 1 year (95% CI: 4.7–22.1%). Only 16% of all patients received WBI in the further course of disease to achieve DC. All other patients could be salvaged via focal treatment.

**Table 1:** Patients charactersitics

Patients characteristics	
number of patients	105
male/female	50/55
patients alive	53
median age (range)	65 years (39–88 years)
patients with previous brain RT	9
patients with metastases in other organs	65
RPA	
class 1	16
class 2	76
class 3	13
lesion characteristics	
median number of BM at treatment (range)	1 (1–6)
median size of treated lesion (range)	3.1 cm (1.3- 7.0 cm)
median size of applicator (range)	2.0 cm (1.5 – 4.0 cm)
median dose (range)	20 Gy (13.4–30 Gy)
suspected incomplete resection in MRI	41
localization of brain metastases	
frontal	33
parietal	25
occipital	26
temporal	18
posterior fossa	15
histology of resected metastases	
NSCLC	50
melanoma	16
breast cancer	13
colorectal carcinoma	9
renal cell carcinoma	8
SCLC	4
upper gastro-intestinal cancer	3
bladder cancer	3
other	11
ovarian cancer/ parotid cancer/ head and neck cancer/ prostate cancer/ sarcoma

IORT did not increase the perioperative toxicity of brain surgery. Thirty-day mortality of the 114 interventions was 5.3%, not related to IORT. All of these patients had been already discharged from hospital after brain surgery and most of them had even received another course of systemic treatment before their death. Five patients died from a sepsis arising either of the genitourinary or respiratory tract and one patient suffered a lethal infarction of the arteria cerebri media 12 days postoperatively. The latter was an 82 year old male patient, treated for a singular symptomatic 2.5 cm left occipital BM from NSCLC. Staging showed no evidence of metastatic disease elsewhere. He had a history of myocardial infarction treated with a coronary stent 14 years before and a pulmonary embolism five years earlier. Anticoagulation with acetylsalicylic acid was paused for 2 weeks in total due to brain surgery. After complete microsurgical resection an IORT with 18 Gy to the surface of the spherical 1.5 cm applicator was performed. The patient was discharged from hospital in good health six days after surgery. Another five days later he was brought to the emergency room after a syncopal collapse with multiple bruises. CT showed signs of an infarction of the left medial cerebral artery. Due to the recent neurosurgery no lysis therapy was performed and patient died one day later.

Six postoperative bleedings (2 revisions), four postoperative formations of hygromas and four wound infections (1 revision) in the area of the craniotomy were observed. One IORT procedure in the posterior fossa had to be terminated prematurely (applied 13.4 Gy of planned 18 Gy) due to the detection of an air embolism, which could be treated without consequential damage for the patient. It was a 79 year old female patient treated for a symptomatic 3.5 cm right sided metastasis in the cerebellum from a malignant melanoma. Surgery was performed in semi-sitting position. In this setting air embolism is a well-known surgical complication. IORT was performed with a spherical 2.5 cm applicator. A dose of 18 Gy on the surface of the applicator was intended. Despite of the reduced applied dose no local failure occurred in the further FU of this patient. Our institutional review of this case found no impact of the IORT on the occurrence of the air embolism. It was assumed that the air was sucked in via a vein affected during the surgical procedure before. After changing the surgical patient positioning to a lateral approach, no further air embolisms were observed during an IORT procedure. In an already published analysis of our center [19] we found no increased toxicity of IORT in the posterior fossa compared to supratentorial lesions.

Median time from surgery to discharge from hospital was 6 days (range 2–41 days). IORT procedure prolonged operation room (OR) time for 25 min in mean (range 15–42 min). Mean operation time including IORT was 150 min (range 97 to 308 min). Mean radiation time was 15:13 min (range 6:00–28:13 min). Fifty-eight patients in this series needed systemic treatment due to additional tumor burden in other organs. Median time to start of systemic treatment after surgery was 24 days (range 1 to 136 days) for these patients. Symptomatic brain necrosis (RN) after IORT was observed and histologically proven in one single case of a lesion in a pre-irradiated area. Two other patients developed radiological signs of RN on FU MRI scans 83 days and 538 days after surgery without any neurological symptoms. These contrast enhancing zones next to the resection cavity diminished without any specific treatment during further FU (observed brain necrosis rate 2.6% /estimated brain necrosis rate of 3.9% at 1 year).

## Discussion

To our knowledge, this is the largest published mono institutional series of patients treated with IORT after resection of BM. Since 2020 more patients were treated with IORT than with SRT after microsurgical resection of BM. This is due to patients´ choices, who find the “one stop shop” character of this treatment very appealing. Nevertheless all patients of the UKA, who are planned for BM resection after decision of the MTB, are offered the option of a postoperative cavity SRT as well as the option of IORT.

The observed LC rate of 90.4% with an estimated LC of 84.2% at 1 year (95% CI: 71.3–91.6%) is as well in line with the smaller IORT series of De Castro et al. and Cifarelli et al. [8, 9] as with postoperative cavity SRT series [3, 20–23] and it stayed unchanged compared to our last publication 2021 [15] after the first 40 treated patients. Remarkably, the high rate of suspected incomplete resections on postoperative MRI of 34.6% did not transfer into worse local control (84.7% (95% CI: 66.5–93.4%) R0 vs. 82.1% (95% CI: 58.9–93.0%) R1; p = 0.606) in univariate analysis. This stands in contrast to the findings of Cifarelli et al. [8], who found this to be the only predictor of LC in their data set. We hypothesize that contrast enhancements on the cavity rim as a reaction of the brain tissue after IORT might blur the distinction between residual disease and IORT related tissue changes (Picture 1).

Patients´ median OS of 18.2 months (range 0.5 -79.0 months) stresses the relevance of LC without relevant treatment associated neurotoxicity in this population. Most patients live long enough to experience the impairments either by local recurrence or by therapy associated neurotoxicity. Taking in account the estimated DC of 47.9% at 1 year (95% CI: 34.7–60.0%) close MRI FU is of uttermost importance in this setting to detect the frequent distant relapses before they become symptomatic and deteriorate patients´ quality of life. With the applied UKA FU policy of three-monthly MRIs we were able to detect all distant brain relapses in an asymptomatic state. Most of them could be salvaged with SRT. Only 16% of our patients required WBI due to multiple BMs and/ or LMD during their whole course of cancer treatment. This is in line with data from BM patients treated with SRT [3, 7, 20]. LMD among our patients with an estimated one- year LMD rate of 10.4% (95% CI: 4.7–22.1%) was not observed more frequently than reported in patient series treated with postoperative SRT with one-year LMD rates ranging between 7 and 30% [20, 24–26]. We published the data of patients treated with SRT after neurosurgical BM resection in our center[7]. In this series the estimated one- year LMD rate was 16% (95%CI 7.3–32.9%). Hence, there is no evidence that the IORT procedure increases the risk of tumor cell spillage to the cerebrospinal fluid by neurosurgery. It cloud even be hypothesized that IORT might decrease the LMD rate, as LMD could be affected negatively by the interval between surgery and SRT of the non-sterilized surgical cavity. To address this question a randomized trial is obligatory.

IORT did not increase the perioperative complications rate of brain surgery. Although our series contains cases with huge metastases up to 7 cm diameter, 12% of the treated patients fitted to RPA class 3 and 13% of the metastases were located in the posterior fossa, the 30-days- mortality and complication rate were within the range described in recent publications of patient cohorts after neurosurgical BM resection with or without IORT [3, 9, 19, 27–30]. The complete IORT procedure prolonged OR time for 25 min in mean including less than 16 min radiation time. This and the limited radiation protection measures, comparable to the protection measures required for the use of fluoroscopy, made it possible for our center to integrate the IORT procedure in our routine workflow for microsurgical BM resections easily.

Median time from surgery to discharge from hospital was 6 days (range 2–41 days) for our patients, which is as long as it is for patients after BM resection without IORT. Dejonckheere et al.[31] compared the mean time to next treatment of patients after BM resection with IORT to patients treated with postoperative SRT to the cavity in a retrospective single center study. They found a significant difference in favor of patients treated with IORT (36 (9 − 94) days) versus patients treated with postoperative SRT (52 (11 − 126) days). We observed a mean time to systemic treatment (TTST) of 31 days (median TTST: 24 days (range 1 -136 days)) in our patient set, which is in line with the results of the quoted study.

Within this retrospective single center study we observed an extremely low rate of RN during FU. With an observed RN rate 2.6% and an estimated RN rate of 3.9% at one year, we report a markedly lower RN rate compared to other published data on focal BM cavity radiotherapy. In the current literature the rates of RN rage from 5 to 25% for patients treated with SRT after resection of BM [3, 20, 32–34]. Our comparatively low RN rate could possibly be explained by the relatively small volume of surrounding brain tissue receiving 10 Gy (V10), due to the steep dose gradient of 50 kV x-rays [15]. V10 is an established risk factor for RN in SRT. In our series the mean applicator size was 2.0 cm (range 1.5–4.0 cm), corresponding to a nominal mean V10 of 6.12 cm3 (range 3.08–35.95 cm3). Taking into account relative biological efficacy (RBE) of low energy X-rays, the corresponding mean V10 (RBE) is 12.97 cm3 (range 4.6–48.94 cm3). This data supports the clinical benefit gained from the steep dose gradient of 50 kV x-ray IORT around the resection cavity already described by other groups [10, 35].

Nevertheless there are some restrictions to the interpretation of our data. As a retrospective study it is subjected to bias due to patient selection and reporting. We treated the majority of our patients within the last two and a half years, which puts a limit to the FU time of this data. Some patients were lost to FU, which is a source of further uncertainty. These effects may be partly balanced by the large number of procedures reported. Thus, the results represent the clinical status of IORT after microsurgical resection of BM, which can be achieved as institutional standard treatment in a university center. Never the less future randomized trials are needed to prove the possible advantages of IORT compared to postoperative SRT scientifically sound, which is also true for the comparison of postoperative stereotactic radiosurgery versus fractionated SRT in the same setting.

## Conclusion

After117 procedures IORT as an institutional standard treatment appears to be a safe and appealing way to perform cavity RT after microsurgical resection of BM with low toxicity and excellent LC. For patients with additional systemic tumor burden IORT in this situation holds the chance for an early start of adjacent systemic therapy. Three-monthly FU with MRI is paramount to detect the frequent distant brain failure (DBF) early. In this setting WBI could be avoided for over 80% of the patients in the further course of disease using SRT as effective salvage therapy for DBF.

### Supplementary Information

Below is the link to the electronic supplementary material.

Supplementary file1 Picture 1: Contrast enhanced MRI FU of a patient with suspected residual disease after microsurgical resection and IORT (20Gy/2.5 cm spherical applicator) of a symptomatic left frontal metastasis of NSCLC and disappearing enhancement 6 month after IORT without further treatment. (Left: preoperative status/ middle: status 24h after surgery and IORT /right: status 6 months after surgery and IORT) (JPG 116 KB)

## Data Availability

The datasets generated during and/or analyzed during the current study are available from the corresponding author on reasonable request.
