# Supplementary figures and images for: Intraoperative radiotherapy after neurosurgical resection of brain metastases as institutional standard treatment– update of the oncological outcome form a single center cohort after 117 procedures
Source: J Neurooncol. 2024 Jul 4;169(1):187–93. doi: 10.1007/s11060-024-04691-6 (PMC11269407; doi:10.1007/s11060-024-04691-6)

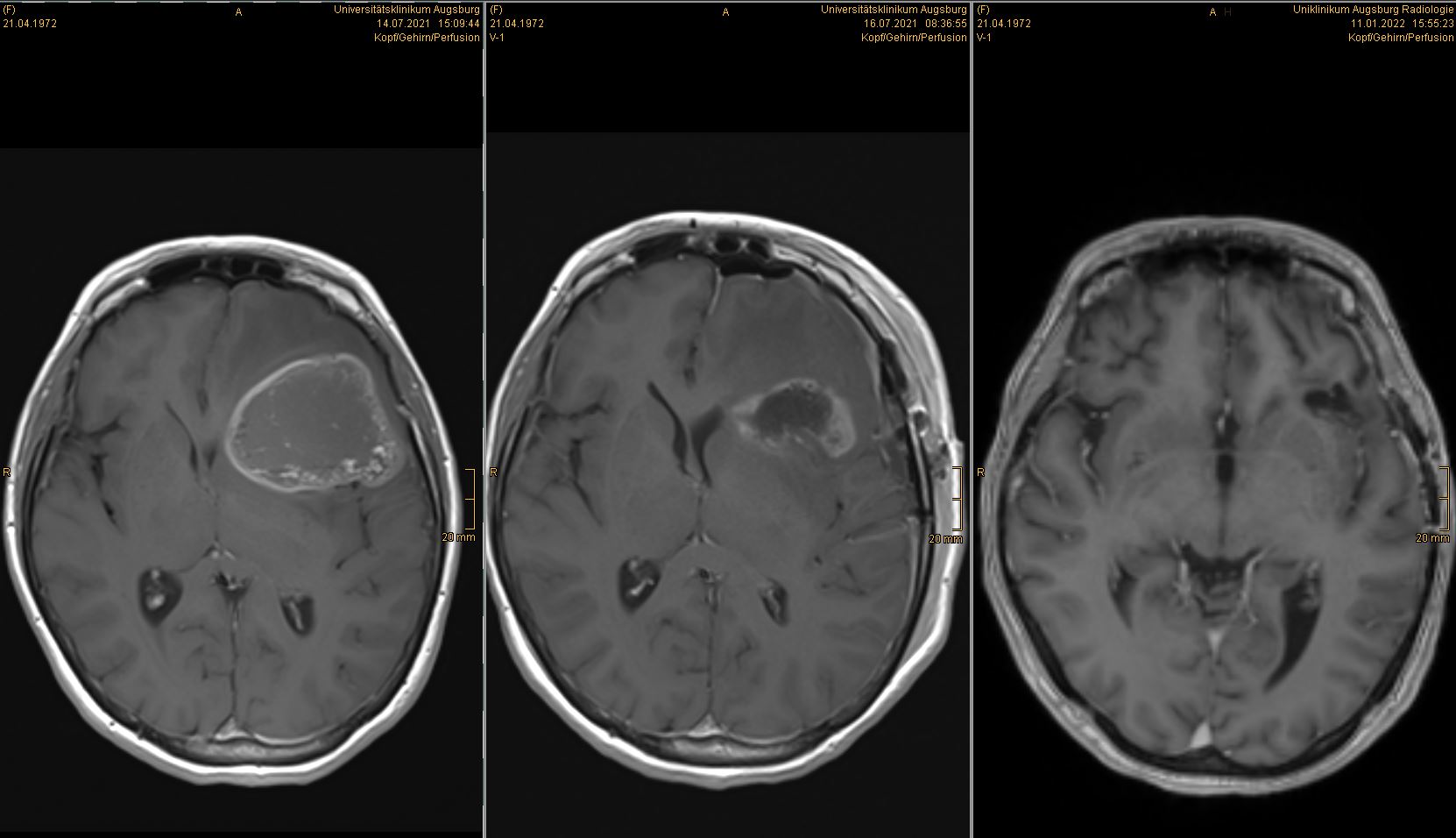

Supplement: Supplementary file 1 — Supplementary file1 Picture 1: Contrast enhanced MRI FU of a patient with suspected residual disease after microsurgical resection and IORT (20Gy/2.5 cm spherical applicator) of a symptomatic left frontal metastasis of NSCLC and disappearing enhancement 6 month after IORT without further treatment. (Left: preoperative status/ middle: status 24h after surgery and IORT /right: status 6 months after surgery and IORT) (JPG 116 KB) [file 11060_2024_4691_MOESM1_ESM.jpg]
